# De novo transcriptome analysis provides insights into the salt tolerance of *Podocarpus macrophyllus* under salinity stress

**DOI:** 10.1186/s12870-021-03274-1

**Published:** 2021-10-25

**Authors:** Lijuan Zou, Taotao Li, Bingbing Li, Jing He, Chunli Liao, Lianzhe Wang, Shouyu Xue, Tao Sun, Xuan Ma, Qinggui Wu

**Affiliations:** 1 Ecological Security and Protection Key Laboratory of Sichuan Province, Mianyang Normal University, Mianyang, 621000 China; 2 College of Life Sciences and Engineering, Henan University of Urban Construction, Pingdingshan, 467036 Henan China; 3 The Environmental Monitoring Station of Chuanshan District, Suining, 629000 China; 4 National Key Laboratory of Crop Genetic Improvement, Huazhong Agricultural University, Wuhan, 430070 China

**Keywords:** *Podocarpus macrophyllus*, Salt stress, Transcriptome, Phytohormone, Transcription factor

## Abstract

**Background:**

Soil salinization is causing ecosystem degradation and crop yield reduction worldwide, and elucidation of the mechanism of salt-tolerant plants to improve crop yield is highly significant. *Podocarpus macrophyllus* is an ancient *gymnosperm* species with a unique environmental adaptation strategy that may be attributed to its lengthy evolutionary process. The present study investigated the physiological and molecular responses of *P. macrophyllus* plants to salt stress by analyzing its photosynthetic system and antioxidant enzyme activity. We also analyzed the differentially expressed genes (DEGs) in *P. macrophyllus* under salt stress using RNA sequencing and de novo transcriptome assembly.

**Results:**

Salt treatment significantly affected the photosynthetic system in *P. macrophyllus* seedlings, which decreased chlorophyll content, altered chloroplast ultrastructure, and reduced photosynthesis. The activities of antioxidant enzymes increased significantly following salt stress treatment. Transcriptome analysis showed that salt stress induced a large number of genes involved in multiple metabolic and biological regulation processes. The transcription levels of genes that mediate phytohormone transport or signaling were altered. K^+^ and Ca^2+^ transporter-encoding genes and the MYB transcription factor were upregulated under salt stress. However, the genes involved in cell wall biosynthesis and secondary metabolism were downregulated.

**Conclusion:**

Our research identified some important pathways and putative genes involved in salt tolerance in *P. macrophyllus* and provided clues for elucidating the mechanism of salt tolerance and the utilization of the salt tolerance genes of *P. macrophyllus* for crop improvement.

**Supplementary Information:**

The online version contains supplementary material available at 10.1186/s12870-021-03274-1.

## Background

Soil salinization and global warming have severe impacts on agricultural production and ecosystems. Global warming will likely exacerbate the risks of soil salinization. Due to rising temperatures that increase water evaporation and the upward movement of salt in soil, global warming aggravates the harm of soil salinity [1]. According to the Food and Agriculture Organization (FAO), over 1.1 billion ha of soil are affected by soil salinization worldwide, which has resulted in a reduction in agricultural production of 20 ~ 46 million ha and enormous annual economic losses [2]. Considering the rapidly growing world population, the global food supply will likely face a serious challenge in the near future. Therefore, improving crop salinity tolerance and the use of salinized soil for agricultural production to ensure food security is of great significance. Understanding the mechanisms of plant responses to soil salinity-induced stress will contribute to improving crop stress resistance and increasing crop yield.

High salinity in soil solutions causes hyperosmotic and ionic toxicity stress and induces secondary stresses, such as oxidative stress, in plants. These stressors cause damage to cellular membranes, proteins, nucleic acids, and the photosynthetic apparatus, which result in growth retardation and metabolic dysfunction [3, 4]. Plants use specialized strategies to cope with salt stress. For example, plants alleviate hyperosmotic stress damage by accumulating osmoprotectants, such as proline, polyamines, glycine-betaine, and sugars, that are synthesized by certain metabolic pathways [5, 6]. For toxic ion stress, primarily Na^+^ and Cl^−^, plants minimize their harmful effects by activating certain ion transporters that squeeze Na^+^ out of cells and/or compartmentalize Na^+^ into vacuoles, such as salt overly sensitive 1 (SOS1), which is a Na^+^/H^+^ antiporter expressed in root epidermal cells that extrudes Na^+^ into the soil [7]. High affinity potassium transporter 1 (HKT1) is a Na^+^/K^+^ co-transporter expressed in xylem parenchyma cells that unloads Na^+^ from xylem sap into xylem parenchyma cells to protect leaves [8, 9]. The ability of transporters to take up K^+^ is critical for maintaining Na^+^/K^+^ homeostasis [3, 8]. Salt stress-induced osmotic stress and ionic stress trigger the excessive accumulation of reactive oxygen species (ROS), which results in oxidative damage to plant cells. The antioxidant system protects plants from oxidative stress damage by detoxifying ROS and maintaining the balance of ROS generation under salt stress [6]. The antioxidant defense system includes enzymatic and non-enzymatic components in plants. The major antioxidant enzymes include superoxide dismutase (SOD), peroxidase (POD), catalase (CAT), ascorbate peroxidase (APX) and glutathione reductase (GR), and non-enzymatic components, such as glutathione (GSH), ascorbic acid, flavonoids, and carotenoids [5, 6, 10]. Previous studies reported that many phytohormones, such as abscisic acid (ABA), ethylene, jasmonates (JA), and salicylic acid (SA), are also associated with signaling and antioxidant defense systems to protect plants exposed to salt stress [7, 11, 12]. Salinity-induced ROS over-generation is a main hindrance of plant physiological and biochemical metabolic activities, which are largely restored via enhancement of the antioxidant defense system that scavenges ROS [5–7].

High-throughput sequencing technology, especially RNA sequencing (RNA-seq), has been widely used to study the molecular basis of plants in response to abiotic stress in recent years [13–15]. De novo transcriptome assembly by RNA-seq enables research on species without a reference genome [16]. RNA-seq was recently used to reveal dynamic changes in the transcriptomic profile of many species, such as *Pinus halepensis* and *Pinus massoniana* [14, 17], *Populus* [18], *Rosa chinensis* [19], halophyte plant *Clerodendrum inerme* (L.) Gaertn [15], and *Suaeda fruticosa* [13], under abiotic stress. *Podocarpus macrophyllus* ([Thunb.] D. Don) is an ancient woody plant that belongs to the genus *Podocarpus* in the subphylum *gymnospermae*. It is widely distributed in East Asia and the Southern Hemisphere [20]. *P. macrophyllus* is used in traditional medicine for the treatment of various diseases and as an ornamental tree and source of timber [20, 21]. Due to its stout trunk and large canopy architecture, it was named “Buddhist pine” in China. As an ancient woody plant, *P. macrophyllus* has a strong adaptability to soil and is tolerant of soil salinity [22, 23]. However, there is little research on the physiology and molecular mechanisms of *P. macrophyllus* in response to abiotic stress, particularly salt stress.

The present study evaluated the salt tolerance of *P. macrophyllus* under different salinity concentrations in soil and investigated the physiological response and gene expression using transcriptome sequencing of *P. macrophyllus* under short-term (3 h NaCl treatment) and long-term (14 days NaCl treatment) salt stress treatments. We also assembled a high-quality de novo transcriptome of *P. macrophyllus*. Our results elucidate the mechanisms underlying the tolerance of *P. macrophyllus* to salinity and provide a valuable genetic resource for further research on *Podocarpus* plants.

## Results

### Morphological and physiological responses to salt stress

**Fig. 1:**
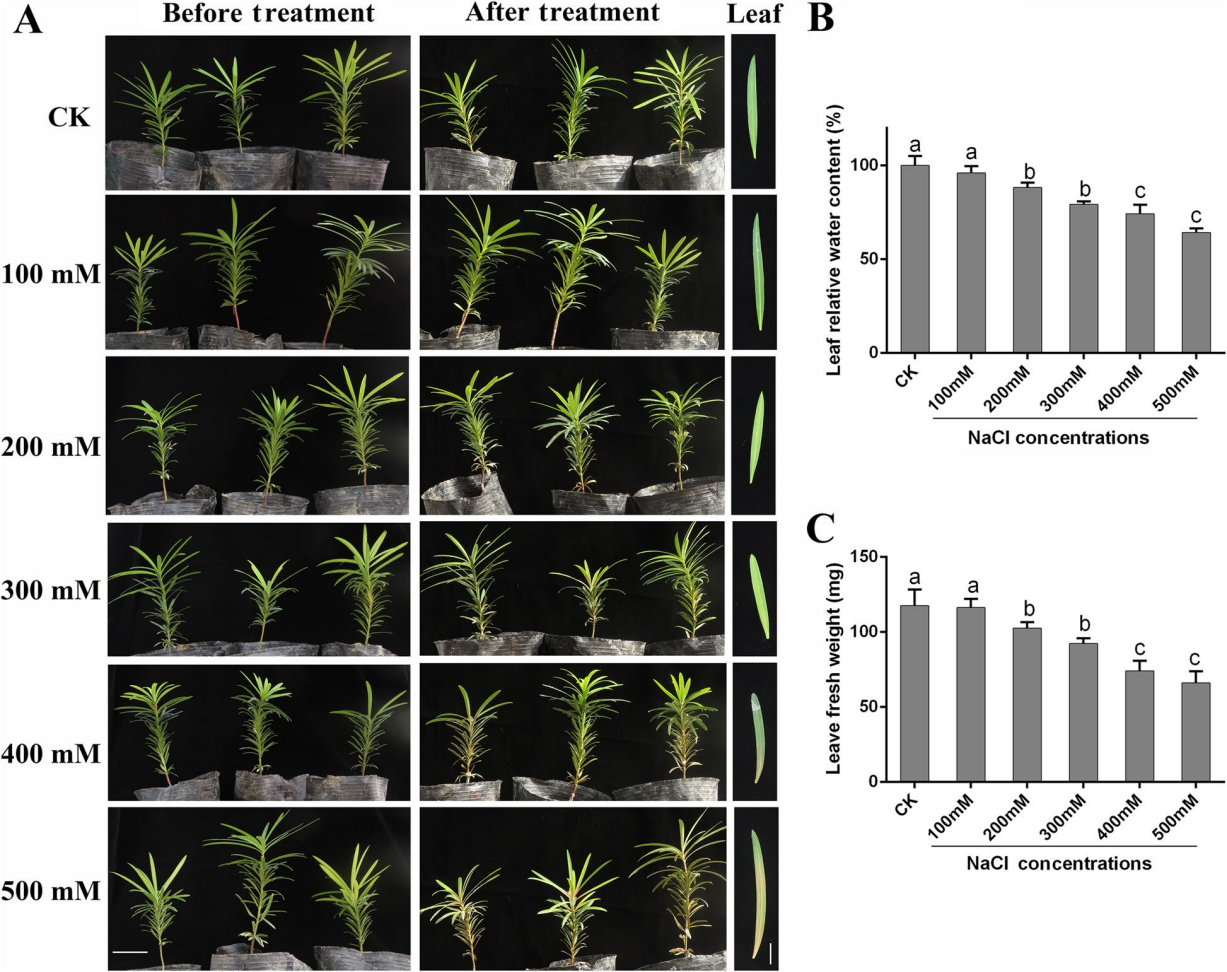
Effects of salt stress treatment with different NaCl concentrations on *P. macrophyllus* seedlings. **A** Phenotype of *P. macrophyllus* seedling shoots under different concentrations of NaCl (0 mM [control, CK], 100 mM, 200 mM, 300 mM, 400 mM and 500 mM) treatment. Left, before treatment, right, after treatment for 14 days. Scale bar = 5 cm. **B** Leaf relative water contents of *P. macrophyllus* under different concentrations of NaCl treatment. **C** Leaf fresh weight of *P. macrophyllus* under different concentrations of NaCl treatment. Data shown are the average mean ± *SE* of three replicates (*n* = 3). Different letters above the bars indicates statistical significance at the *P* < 0.05 level among different concentrations of NaCl treatment according to Tukey’s test

To evaluate the salt tolerance of *P. macrophyllus*, we treated the seedlings with six concentrations of a NaCl solution (0 mM [control, CK], 100 mM, 200 mM, 300 mM, 400 mM, and 500 mM) in soil for 2 weeks. No obvious phenotypic variation was observed in seedling shoots under low NaCl concentration (≤ 200 mM) treatments. For the 300 mM NaCl treatment, the seedling leaves turned yellow and curled, and a severely stressed phenotype was observed for the 400 mM and 500 mM NaCl treatments (Fig. 1A). The leaf relative water (LRW) content showed a significant decrease following treatment with ≥200 mM NaCl compared to the CK and 100 mM NaCl treatment groups (Fig. 1B). The fresh weight measurements of the leaves validated the results of the LRW analysis (Fig. 1C).

### Effects of salt stress on the antioxidant system

**Fig. 2:**
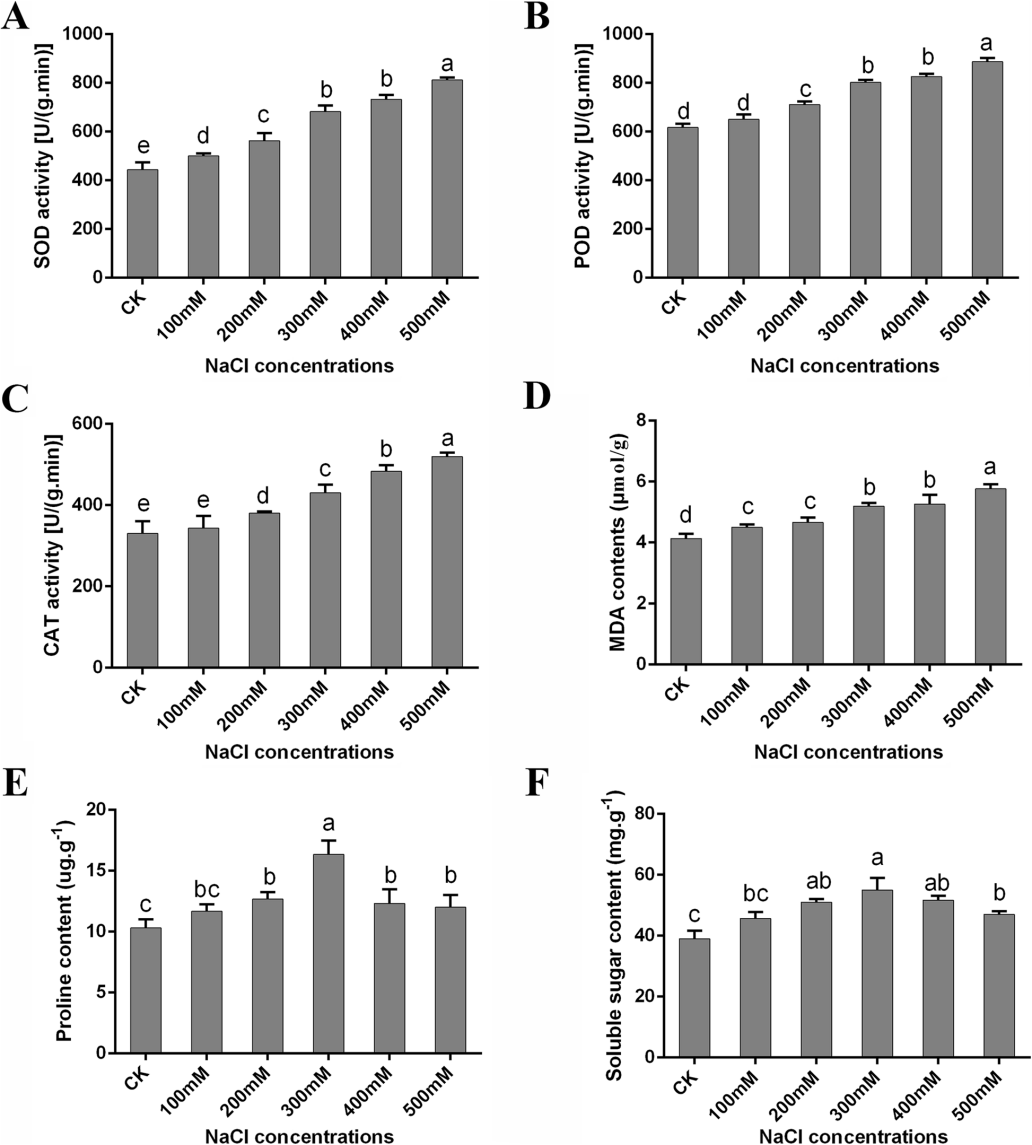
Effects of salt stress on (**A-C**) the antioxidant enzymes acticities, **D** MDA concentration, **E** Proline concentration and **F** Soluble sugar content in leaves of *P. macrophyllus*. **A** Superoxide dismutase (SOD), **B** Peroxidase (POD), and **C** Catalase (CAT) activities in leaves of *P. macrophyllus* seedlings under different concentrations of NaCl treatment for 14d. Data shown are the average mean ± *SE* of three replicates (*n* = 3). Different letters above the bars indicates statistical significance at the *P* < 0.05 level among different concentrations of NaCl treatments according to Tukey’s test

The activities of three antioxidant enzymes (SOD, POD, and CAT) were determined in leaves from different NaCl treatment groups (Fig. 2A–C). Compared to the CK, the activities of SOD, POD and CAT increased significantly under high-concentration salt treatments (≥ 200 mM) (Fig. 2B and C). Oxidative damage in the leaves of *P. macrophyllus* plants was assessed by measuring the concentration of malondialdehyde (MDA). The concentration of MDA was significantly increased under salt treatment compared to the CK (Fig. 2D). Compared to the CK, the contents of proline and soluble sugar in the leaves of *P. macrophyllus* were significantly increased in the groups treated with ≥200 mM NaCl and reached a maximum in the 300 mM treatment group (Fig. 2E and F). These results suggest that 300 mM NaCl induced a complete physiological and biochemical response to salinity stress in *P. macrophyllus*.

### Effects of salt stress on the photosynthetic system

**Fig. 3:**
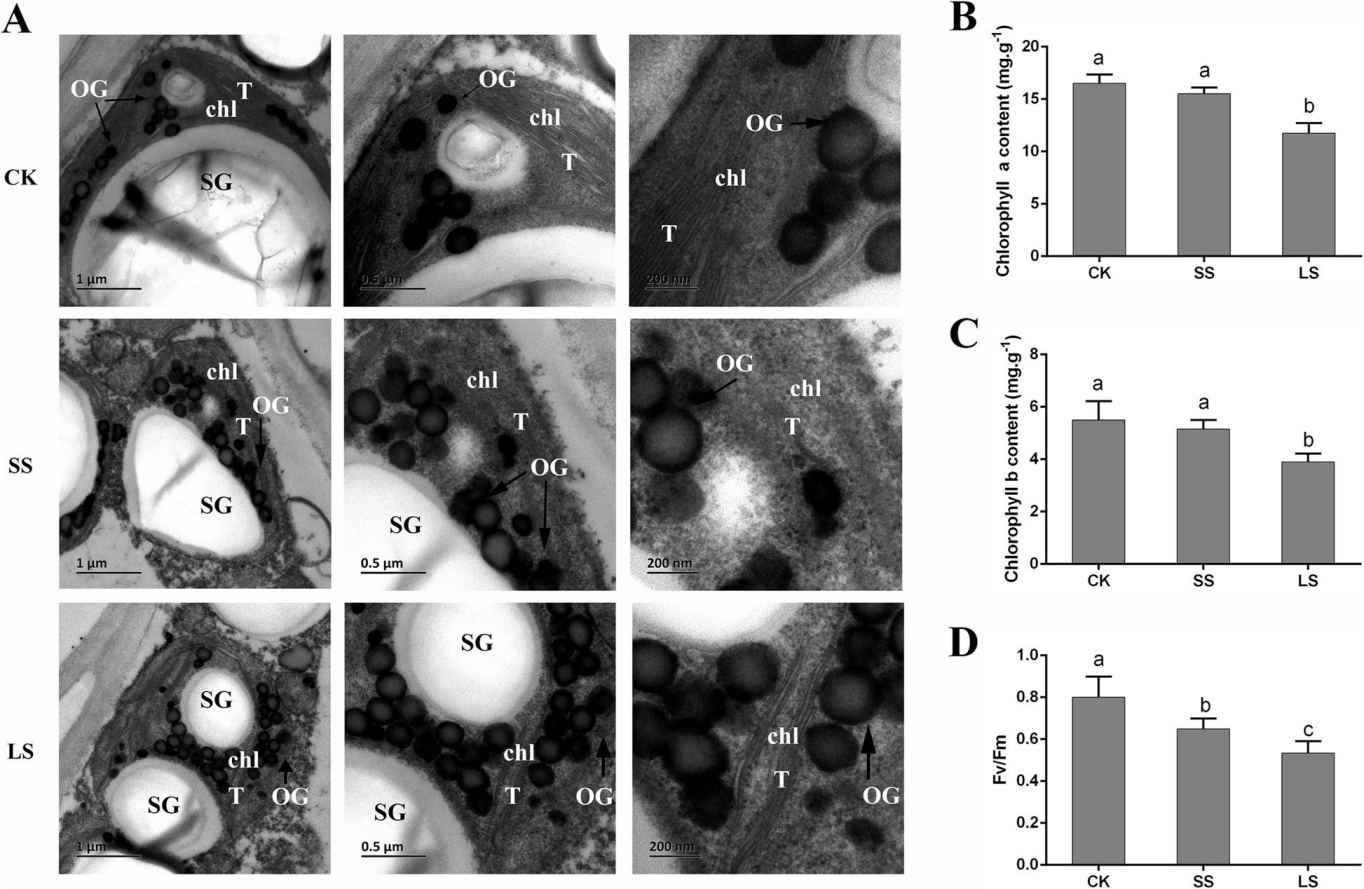
Effects of salt stress on the photosynthetic system of *P. macrophyllus*. **A** Ultrastructure of *P. macrophyllus* chloroplasts with or without (CK) salt stress treatment (300 mM NaCl). Scale (1 μm, 0.5 μm and 0.2 μm). SS, short-term salt treatment. LS, long-term salt treatment. T, OG, chl and SG indicates the thylakoid lamellae, osmiophilic globule, chloroplasts and starch grain, respectively. **B** The contents of chlorophyll *a* and **C** chlorophyll *b* in leaves of *P. macrophyllus* under SS and LS salt stress treatment. **D** The maximum quantum yield (Fv/Fm) in leaves of *P. macrophyllus* under SS and LS salt stress treatment. Data shown are the average mean ± *SE* of three replicates (*n* = 3). Different letters above the bars indicates statistical significance at the *P* < 0.05 level among different concentrations of NaCl treatments according to Tukey’s test

To study the effects of salt stress on the photosynthetic system of *P. macrophyllus*, we measured the chlorophyll content and maximum quantum yield (Fv/Fm), which is a chlorophyll *a* fluorescence parameter, and observed the ultrastructure of *P. macrophyllus* chloroplasts with and without salt stress (Fig. 3). *P. macrophyllus* seedlings treated with 300 mM NaCl in the short-term (SS) and long-term (LS) were used. Observations of chloroplast ultrastructure showed that thylakoid lamellae were neatly stacked, and the presence of starch grains (SG) with an oval or elliptical shape and an intact chloroplast structure in the CK group. Under salt stress, the stroma were vacuolized or broken, and a large number of osmiophilic granules (OGs) were observed in the chloroplasts (Fig. 3A). This observation indicated that the chloroplast structure was severely damaged following salt stress. The Fv/Fm was significantly lower following SS and LS salt stress compared to the CK (Fig. 3B). Chlorophyll *a* and chlorophyll *b* contents were also significantly reduced following LS salt stress compared to the CK and SS groups (Fig. 3C and D).

### RNA sequencing, de novo assembly, and transcriptome annotation

To investigate the molecular basis of *P. macrophyllus* response to salt stress, we performed transcriptomic analysis of the leaves of *P. macrophyllus* seedlings following SS or LS salt stress with 300 mM NaCl using RNA-seq on the Illumina NovaSeq 6000 platform. Three biological replicates were sequenced for each treatment group and the control, and 65.15 Gb data were obtained from nine cDNA libraries. After trimming the low-quality reads and adapters, we obtained 217,582,115 clean reads with a Q30 higher than 94.27% (Supplementary Table 1). There is no available reference genome sequence for the alignment of *P. macrophyllus* RNA-seq reads. Therefore, we assembled the transcriptome de novo using Trinity software [14]. A total of 156,544 transcripts and 65,555 unigenes were obtained, with an N50 length of 1617 bp and an average length of 988 bp (Table 1). Unigenes with lengths of 300–500 bp, 500–1000 bp, 1000–2000 bp, and > 2000 bp accounted for 45.4, 26, 15.8, and 12.8%, respectively (Supplementary Fig. 1; Table 1), and 18,276 unigenes had lengths > 1000 bp.

**Table 1:** Summary of de novo assembled transcriptome of *P. macrophyllus*

Category	Length Range				Total number	Total length (bp)	N50 (bp)	Mean length
	300-500 bp	500-1000 bp	1000-2000 bp	> 2000 bp				
Transcript	38,007(24.28%)	32,620(20.84%)	38,678(24.71%)	47,239(30.18%)	156,544	252,309,972	2496	1611.75
Unigene	29,764(45.40%)	17,065(26.03%)	10,358(15.80%)	8368(12.76%)	65,555	64,780,556	1617	988.19

**Fig. 4:**
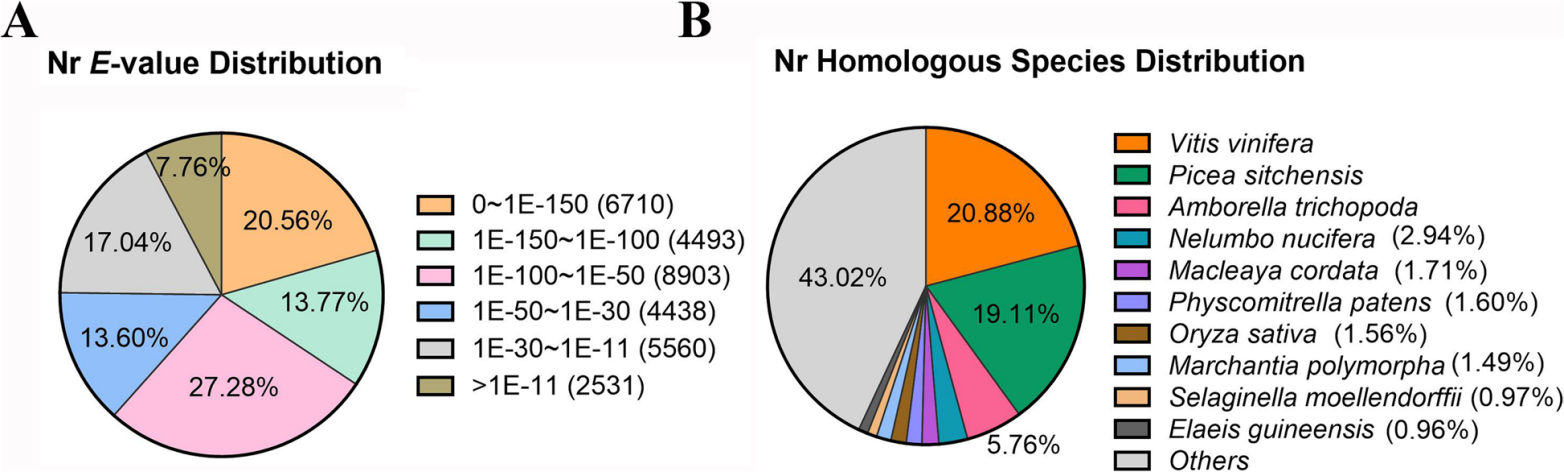
Characteristics of homology search of unigenes against NCBI non-redundant (Nr) database in *P. macrophyllus*. **A** The *E*-value distribution of BLAST hits for each unique sequence with a cut-off *E*-value of 1E-5. **B** Species distribution of the top BLAST hits for each unigene with a cut-off *E*-value of 1E-5

The assembled unigenes were annotated using the NCBI non-redundant (Nr), Swiss-Prot, Gene Ontology (GO), Kyoto Encyclopedia of Genes and Genomes (KEGG), Protein family (Pfam), Clusters of Orthologous Groups (COG), evolutionary genealogy of genes: Non-supervised Orthologous Groups (eggNOG), and EuKaryotic Orthologous Groups (KOG) databases using the BLAST algorithm with an *E*-value < 1.0 × 10^− 5^. A total of 35,542 unigenes (54.22%) were matched to known genes at least once in the above databases. A total of 33,480 (51.1%) and 32,635 (49.8%) unigenes were the best hits in the eggNOG and Nr databases, respectively, followed by 26,071 (39.8%) in the Pfam database, 23,037 (35.1%) in the Swiss-port database, 19,188 (29.3%) in the GO database, 14,546 (22.2%) in the KEGG database, and 11,937 (18.2%) in the COG database (Table 2). The *E*-value distribution of annotation based on the Nr database showed that a large number of unigenes (61.61%) had *E*-values less than 1E^− 50^ (Fig. 4A), which indicated strong homology. The unigene sequences were matched against the Nr database with the highest match score to the gene sequences from *Vitis vinifera* (20.88%), *Picea sitchensis* (19.11%), *Amborella trichopoda* (5.76%), *Nelumbo nucifera* (2.94%), *Macleaya cordata* (1.71%), *Physcomitrella patens* (1.60%), *Oryza sativa* (1.56%), *Marchantia polymorpha* (1.49%), *Selaginella moellendorffii* (0.97%), and *Elaeis guineensis* (0.96%) (Fig. 4B).

**Table 2:** Statistics of the annotated unigenes number in *P. macrophyllus*

Database	Annotated Number	300 ≤ length < 1000	Length ≥ 1000
COG annotation	11,937	5220	6717
GO annotation	19,188	10,918	8270
KEGG annotation	14,546	7796	6750
KOG annotation	22,717	12,694	10,023
Pfam annotation	26,071	12,572	13,499
Swissport annotation	23,037	11,548	11,489
eggNog annotation	33,480	18,218	15,262
Nr annotation	32,635	17,251	15,384
All annotated	35,542	19,773	15,769

**Fig. 5:**
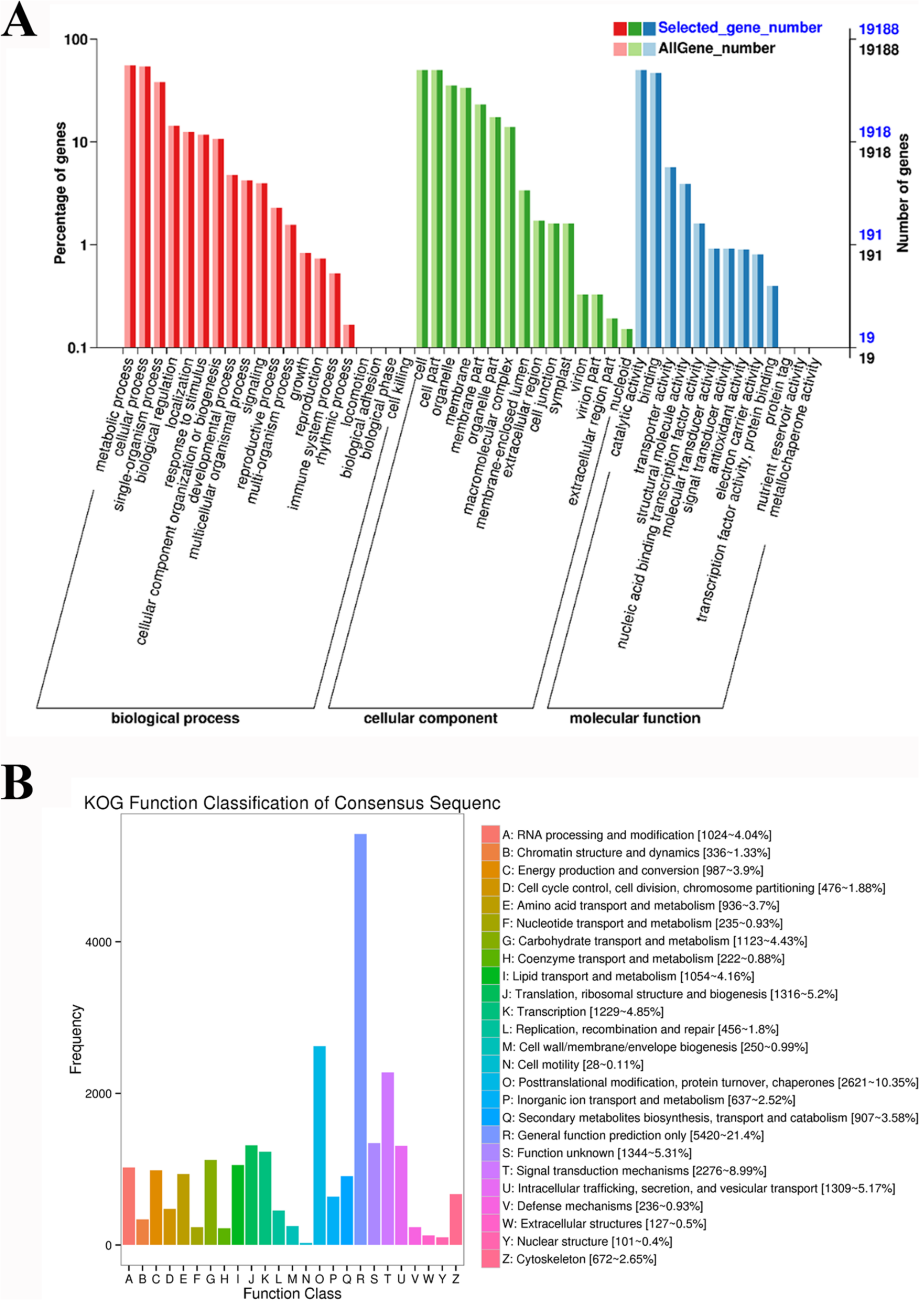
**A** Gene ontology (GO) and **B** EuKaryotic Orthologous Groups (KOG) classification of all unigenes in *P. macrophyllus*. GO classified as biological process, cellular component, and molecular function

A total of 19,188 unigenes were the best hits in the GO database and were enriched in 48 GO terms that were classified as biological process, cellular component, and molecular function. Under biological process, most of the unigenes were enriched in “metabolic process” (10,615), “cellular process” (10,400), “single-organism process” (7274), “biological regulation” (2748), “localization” (2384), “response in stimulus” (2263), and “cellular component organization or biogenesis” (2039) terms, which accounted for 55.3, 54.2, 37.9, 14.3, 12.4, 11.8, and 10.6%, respectively. The other processes accounted for less than 10%. For the cellular component category, the large subcategories were “cell” (9654; 50.3%), “cell part” (9592; 50%), “organelle” (6792; 35.4%), and “membrane” (6428; 33.5%). For molecular function, most unigenes were enriched in “catalytic activity” (9563; 49.8%) and “binding” (9017; 47%) (Fig. 5A).

The KOG tool is a eukaryote-specific version of the COG database for identifying orthologs or paralog proteins [24]. We obtained a total of 22,717 unigenes annotated by the KOG database and divided these unigenes into 25 categories (Fig. 5B). The following top 10 classes with unigenes greater than 1000 were identified: (R) “General function prediction only” (5420, 21.4%), (O) “Posttranslational modification, protein turnover, chaperones” (2621, 10.35%), (T) “Signal transduction mechanisms” (2276, 8.99%), (S) “Function unknown” (1344, 5.31%), (J) “Translation, ribosomal structure, and biogenesis” (1316, 5.2%), (U) “Intracellular trafficking, secretion, and vesicular transport” (1309,5.17%), (K) “Transcription” (1229, 4.85%), (G) “Carbohydrate transport and metabolism” (1123, 4.43%), (I) “Lipid transport and metabolism” (1054, 4.16%), and (A) “RNA processing and modification” (1024, 4.04%) (Fig. 5B).

### Analysis of differentially expressed genes (DEGs) and gene co-expression clusters

**Fig. 6:**
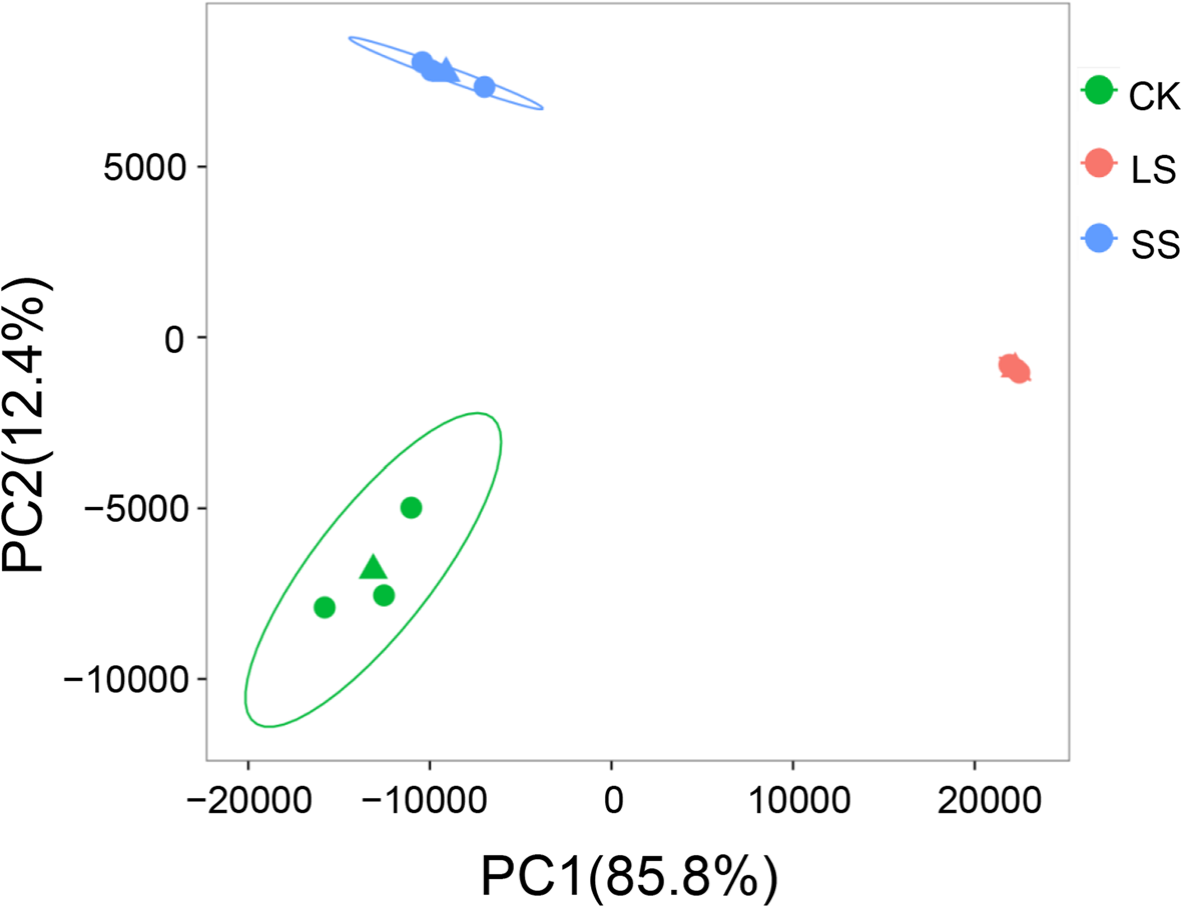
Principal component analysis of transcriptomes of *P. macrophyllus* under salt stress and control. Green circles represent CK, red circles represent LS, and blue circles represent SS. For each treatment group, three biological replicates are shown. Triangle indicates the average of the three biological replicates

**Fig. 7:**
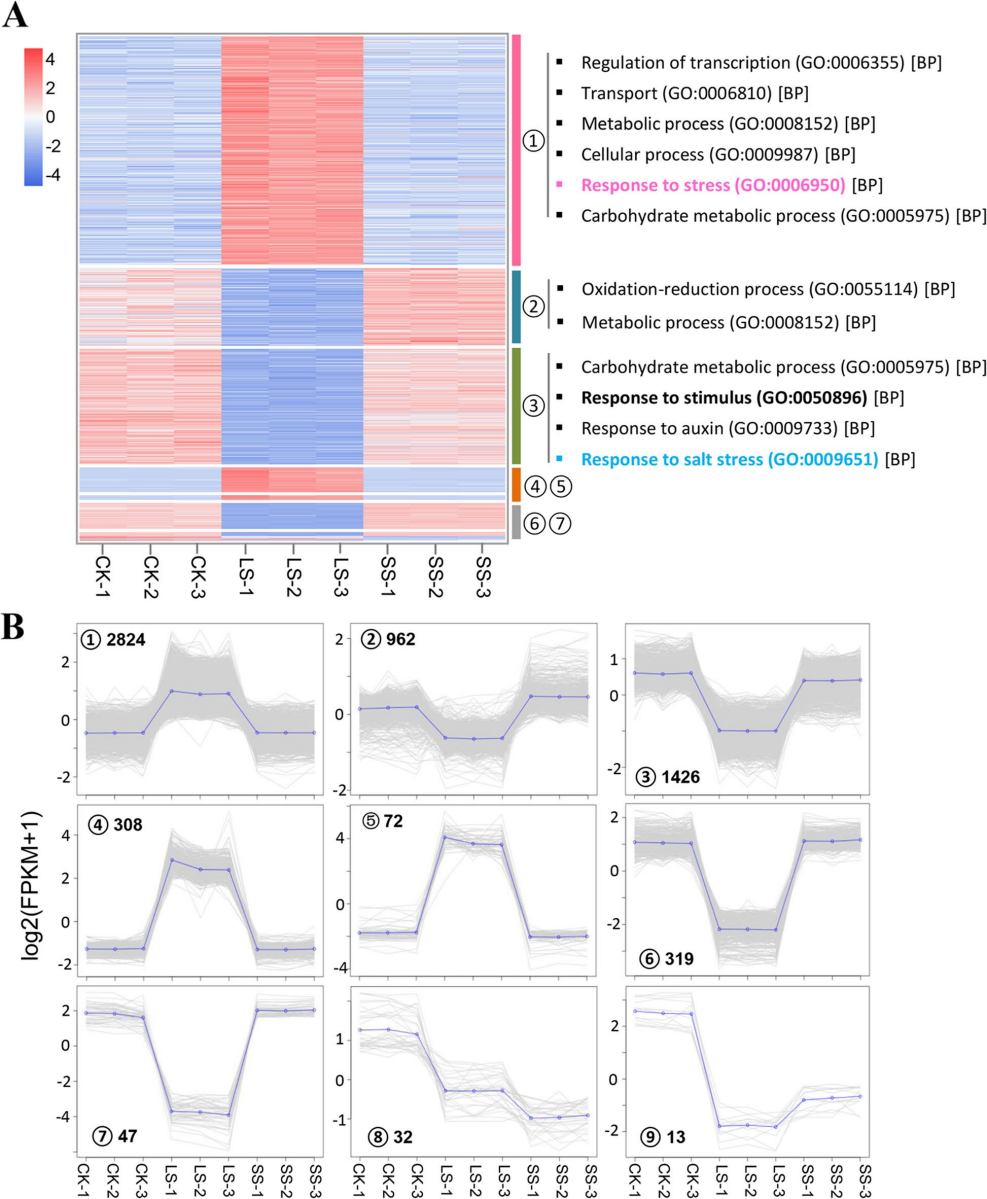
Heatmap and gene co-expression clusters analysis of differentially expressed genes in *P. macrophyllus* under SS and LS salt stress. **A** Heatmap and GO terms that of the enriched in gene co-expression clusters. **B** Gene expression patterns and gene numbers of the different gene co-expression clusters

We aligned the RNA-seq reads of each treatment group and control on the de novo assembled reference transcriptome of *P. macrophyllus*, and the average mapping rate was 84.27% (Supplementary Table 1). There was a high correlation between the three biological replicates of each treatment or control (Pearson’s correlation coefficient was approximately 0.96–1.0) (Supplementary Fig. 2). Principal component analysis (PCA) showed a distinct transcriptome characteristic between the CK and SS and LS groups, with PC1 presenting approximately 86% variation, and the LS was distally apart from the CK and SS groups (Fig. 6). This result suggests that long-term salt stress had a significant effect on transcriptome-wide gene expression in *P. macrophyllus*. DEGs (|log2(fold change)| > 1, FDR < 0.01) were identified in three comparisons (CK vs. LS, CK vs. SS, and SS vs. LS) using DESeq2 software. A total of 6005 DEGs in the three comparisons were divided into nine clusters of gene co-expression patterns (Fig. 7). Gene expression levels were significantly upregulated in Clusters 1, 4 and 5 in the LS group compared to the CK and SS groups, with 2824, 308, and 72 upregulated genes, respectively (Supplementary Dataset 1). GO analysis showed that these genes were involved in the regulation of transcription, transport, metabolic processes, cellular processes, and responses to stress. Seventy-two genes in Cluster 5 had the highest expression levels in the LS group compared to the CK or SS groups (Fig. 7B). These genes may play important roles in improving tolerance to salt or other abiotic stresses in *P. macrophyllus*. The genes in Cluster 2 (962), Cluster 3 (1426), Cluster 6 (319), and specifically Cluster 7 (47) were significantly downregulated in the LS group compared to the CK and SS groups (Supplementary Dataset 2). These genes were involved in the oxidation-reduction process, metabolic process, carbohydrate metabolic process, response to stimulus and auxin, and salt stress (Fig. 7A).

**Fig. 8:**
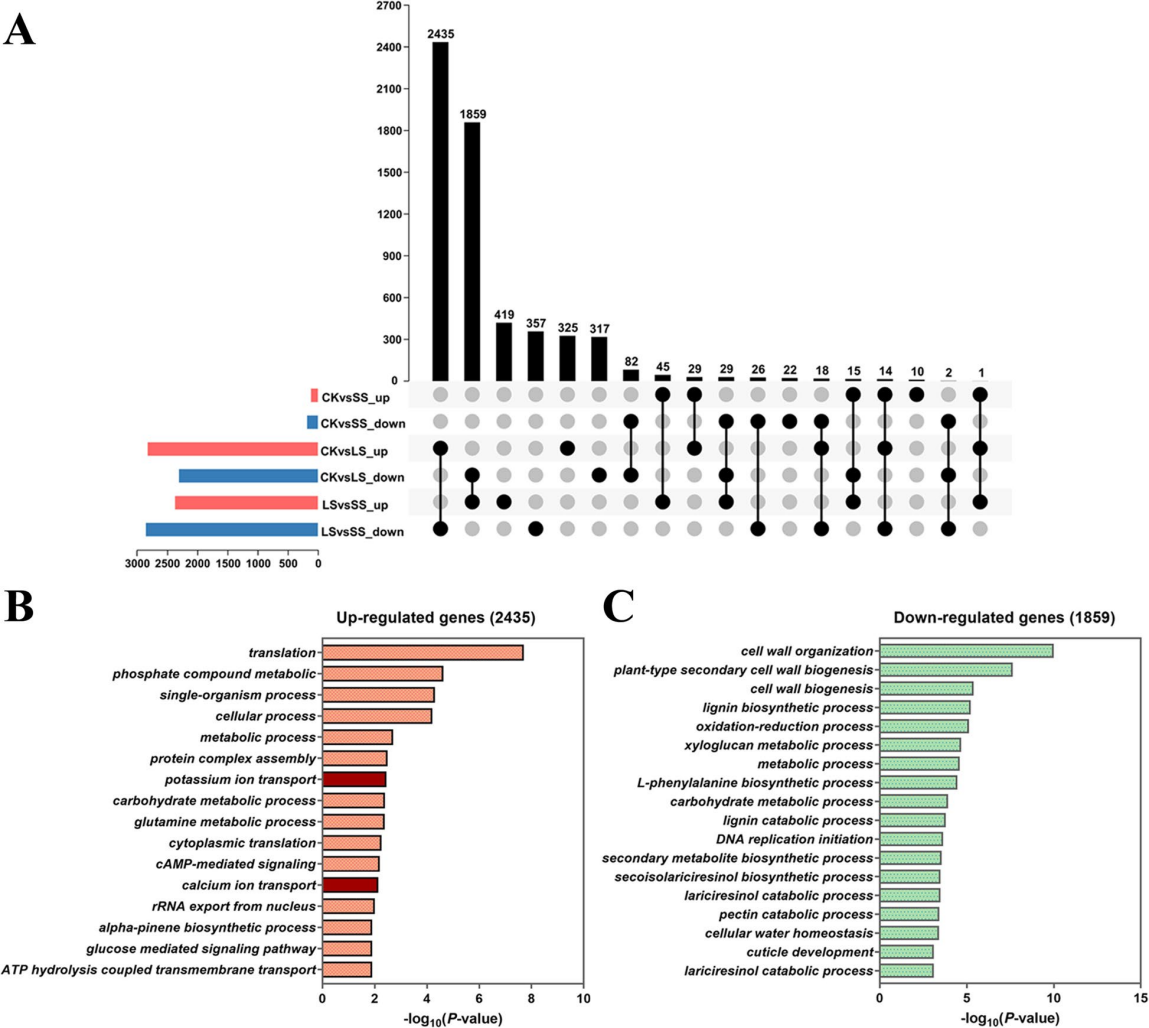
Upsetplots and GO enrichment analysis of the overlapped DEGs in *P. macrophyllus* under SS and LS salt stress. **A** Upsetplots of the overlapped DEGs among multiple comparisons in *P. macrophyllus* under SS and LS salt stress. **B** GO enrichment analysis of upregulated genes and (**C**) downregulated genes

To further study the characteristics of gene expression and dissect the mechanism of salt-tolerance in *P. macrophyllus* under salt stress, we analyzed the DEGs identified in the three comparisons (CK vs. LS, CK vs. SS, and LS vs. SS) and the overlapping DEGs between them. A total of 293 DEGs were identified in CK vs. SS, including 114 upregulated and 179 downregulated genes. A large number of DEGs (5126) were identified in CK vs. LS, including 2822 upregulated and 2304 downregulated genes. The number of DEGs in LS vs. SS was comparable with CK vs. LS, but the downregulated genes were more than those upregulated genes in LS vs. SS. As shown in Fig. 8A, 29 genes were commonly upregulated and 82 genes were commonly downregulated in CK vs SS and CK vs LS. Notably, we observed a large number of common DEGs in CK vs. LS that overlapped with LS vs. SS. Among these genes, 2435 upregulated genes in CK vs. LS overlapped with the downregulated genes in LS vs. SS and showed a higher expression level in the LS group than the CK and SS groups. Their expression patterns were similar to the genes of Clusters 1, 4, and 5. Conversely, there were 1859 downregulated genes in CK vs. LS that overlapped with the upregulated genes in LS vs. SS, and the expression pattern of these genes was the same as the expression in Clusters 2, 3, 6, and 7 (Fig. 8A).

GO enrichment analysis was performed on the overlapping genes between CK vs. LS and LS vs. SS. The upregulated genes (2435) in CK vs. LS were primarily enriched in several processes, such as translation (GO:0006412), phosphate-containing compound metabolic process (GO:0006796), single-organism process (GO:0044699), cellular process (GO:0009987), metabolic process (GO:0008152), and protein complex assembly (GO:0006461). Notably, many pathways involved in ion transport and primary metabolism were significantly enriched, such as potassium ion transport (GO:0006813), calcium ion transport (GO:0006816), carbohydrate metabolic process (GO:0005975), and glutamine metabolic process (GO:0006541) (Fig. 8B). The downregulated genes (1859) were primarily enriched in cell wall organization (GO:0071555), plant-type secondary cell wall biogenesis (GO:0009834), cell wall biogenesis (GO:0042546), lignin biosynthetic process (GO:0009809), oxidation-reduction process (GO:0055114), xyloglucan metabolic process (GO:0010411), metabolic process (GO:0008152), and many biosynthetic metabolic processes (Fig. 8C). These results indicated that most of the genes involved in K^+^ and Ca^2+^ transport were significantly upregulated, and the genes involved in secondary metabolic and biosynthetic processes were significantly downregulated in the leaves of *P. macrophyllus* following salt stress.

### Responses of transcription factors, phytohormones, and ion transporters to salt stress

**Fig. 9:**
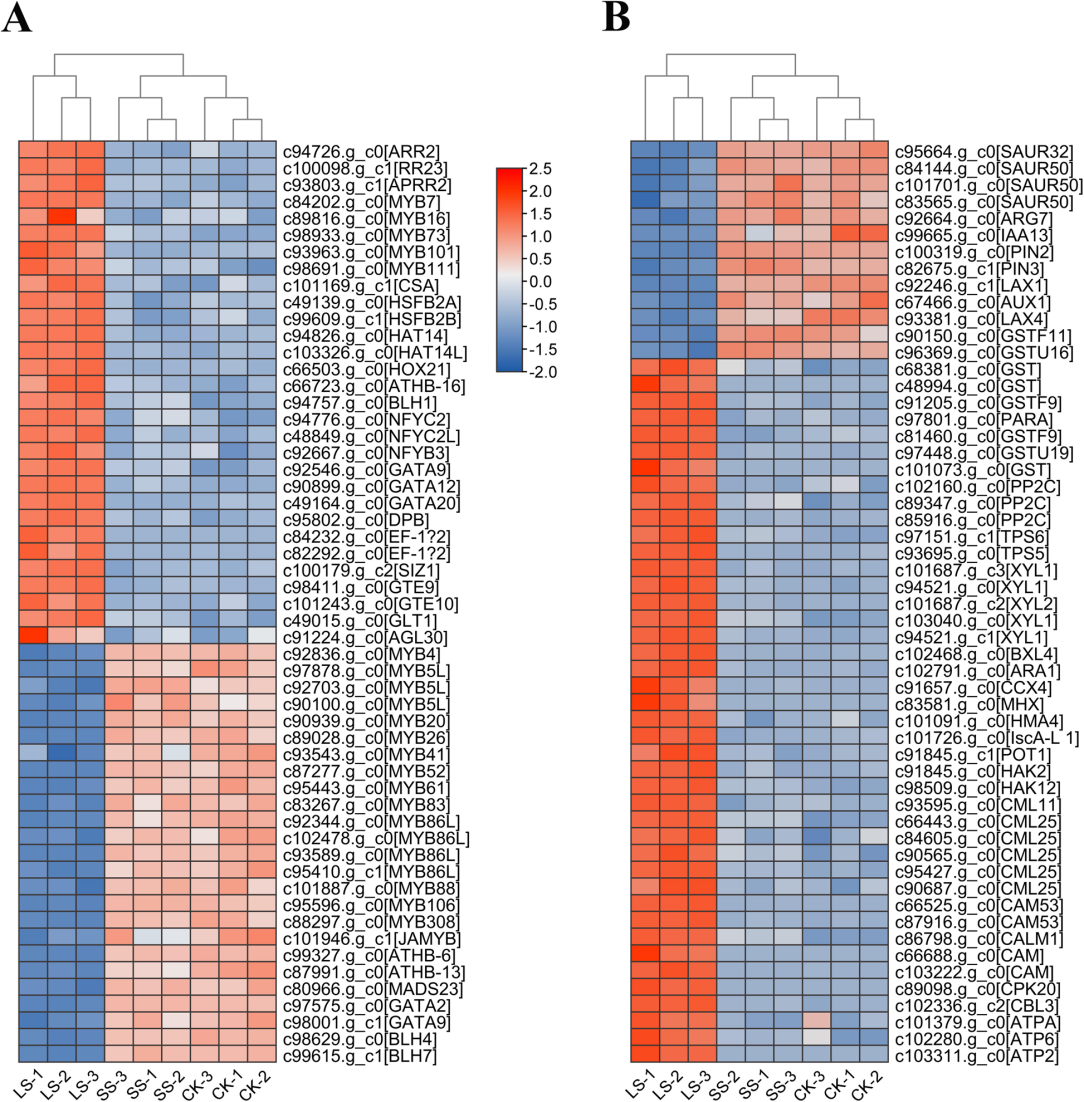
Heatmaps of salt stress responsive genes in *P. macrophyllus*. **A** Transcription factors; **B** Ion transporters and phytohormones signaling related genes

To validate the accuracy of the RNA-seq data of *P. macrophyllus* following salt stress, we randomly selected 12 candidate DEGs (2 upregulated and 10 downregulated genes) and measured their expression levels using quantitative real-time PCR (qRT–PCR) with specific primers (Supplementary Table 2). The results showed that the expression patterns of these genes were highly consistent with the RNA-seq data (Supplementary Fig. 3), which suggested that the RNA-seq data had high reliability, and the DEGs identified in the leaves of *P. macrophyllus* following salt stress were suitable for further analysis. Transcription factors (TFs) are crucial for plant growth and development and plant responses to environmental stimuli or abiotic stress. A total of 2402 TF members were predicted in the assembly transcriptome of *P. macrophyllus*, and the RLK-Pelle, C2H2, AP2/ERF, CAMK, bHLH, MYB-related, MYB, and C3H families of TFs accounted for greater than 60 members (Supplementary Fig. 4). A total of 73 TF members were identified in DEGs (42 upregulated and 31 downregulated) in CK vs. LS and SS vs. LS with well-defined annotations, including 30 MYB TF genes (10 upregulated and 20 downregulated), 11 homeobox domain TFs (6 upregulated and 5 downregulated), 6 GATA TFs (3 upregulated and 3 downregulated), 5 trihelix TFs (upregulated), 5 ARR TFs (upregulated), 4 NF-YC/B TFs (upregulated), 3 MADS-box TFs (1 upregulated and 2 downregulated), 2 heat shock factors (HSF, upregulated), and other TFs (Fig. 9A, Supplementary Dataset 3). We also observed that many phytohormone transport- or synthesis-related genes in the DEGs were identified in the leaves of *P. macrophyllus* following salt stress, such as auxin transporter PINs and AUX1/LAX and auxin-responsive protein SAURs (Fig. 9B), abscisic acid (ABA) receptor PYLs, and gibberellin (GA) receptor GID1. A large number of genes related to auxin transport or signaling were downregulated, and ABA receptor PYLs and GA receptor GID1 were upregulated (Fig. 9B, Supplementary Fig. 5). Nine glutathione S-transferase genes (GSTs), which are involved in glutathione metabolism (ko00480), were identified among the DEGs, and the number of upregulated genes was more than three times of the number of downregulated genes (Fig. 9B). Many genes involved in starch and sucrose metabolism (ko00500), such as trehalose-6-phosphatase synthase (TPS) and alpha-xylosidase (XYLs), were significantly upregulated in *P. macrophyllus* following salt stress, which was highly consistent with the findings of GO enrichment analysis (Fig. 8B) and indicates that carbohydrate metabolism is very important for *P. macrophyllus* during the salt stress response. Notably, many potassium transporters (HAKs), sodium/calcium exchangers (CCX/MHX), calcium-binding protein (CMLs), calmodulin-related protein (CaMs), calcium-dependent protein kinase (CPK), and calcineurin B-like protein (CBL) were significantly upregulated following salt stress (Fig. 9B), which further confirmed the results of GO enrichment analysis of the upregulated genes in CK vs. LS (Fig. 8B). The uptake and transport of K^+^ and Ca^2+^, which maintain Na^+^/K^+^ homeostasis, may be critical for *P. macrophyllus* resistance to salt stress.

## Discussion

Transcriptional regulation, morpho-physiological responses and biochemical metabolism processes play critical roles in plant responses to environmental stimuli [3, 4, 7, 11]. Salt stress is a major abiotic stress that severely endangers plant growth and development and causes a reduction in agricultural production [1, 3, 4]. A large number of studies focused on the mechanism of salt signaling perception and the transcriptional regulation pathway of model plants and staple crops [25–27]. However, investigations of halophyte plants or the plants with high salt tolerance are highly important to elucidate the molecular basis of salt tolerance and identify candidate genes to improve crops. As an ancient *gymnospermae* species, *P. macrophyllus* formed a unique adaptation mechanism to various environmental stimuli because of its lengthy evolutionary process [20]. Therefore, it is very important to investigate the molecular mechanism of its acclimation to the environment and identify novel stress tolerance genes for use as genetic resources to improve the stress tolerance of crops [28].

### *P. macrophyllus* could rapidly acclimate to salinity stress at physiological and transcriptome levels

Oxidative stress has a major detrimental effect on plants due to salinity-induced excessive ROS generation. Plants with high resistance to abiotic or biotic stress generally have a high antioxidative ability and rapid responses to oxidative stress [3, 5, 6]. A high antioxidative ability confers plants stable adaptation to adverse environments and improves their tolerance to stress [4]. Plants scavenge excess ROS produced by salt stress-induced oxidative stress primarily via antioxidant enzymes. The activities of SOD, POD, and CAT in the leaves of *P. macrophyllus* plants were significantly increased under salt stress. At the transcriptome level, many genes involved in the oxidation-reduction process were significantly enriched in DEGs under stress conditions. Plants mitigate the damage caused by oxidative and osmotic stress from increased salinity by producing osmoprotectants via various metabolic pathways. The amounts of soluble sugar and proline were significantly increased in our study due to salt stress. A large number of genes involved in many metabolic pathways, such as the carbohydrate, glutamine, and xyloglucan metabolic pathways, were significantly enriched in DEGs identified in the *P. macrophyllus* transcriptome following salt stress. These results indicated that salt stress induced the expression of genes involved in metabolic pathways and protected plant cells from the harmful effects of high salinity via regulation of the metabolic process. We found that many polysaccharide synthase genes, such as TPS and XYLs, were significantly upregulated following salt stress conditions. Previous studies showed that overexpression of the TPS gene in rice or tomatoes enhanced tolerance to salt or drought stress [29, 30], which was further confirmed in our study.

A large number of DEGs were identified in the transcriptome of *P. macrophyllus* leaves under salt stress treatment. GO enrichment showed that these genes were involved in various biological processes, such as protein translation, multiple biosynthetic or catabolic processes, carbohydrate metabolism, potassium ion transport, and calcium ion transport. The ability to uptake and transport K^+^ and Ca^2+^ is critical for plants to maintain Na^+^/K^+^ homeostasis under salt stress. Ca^2+^ also plays an important role in Na^+^ sensing and signaling in plant cell membrane systems [3, 7, 25]. The present study found that many genes encoding CMLs and CaMs were significantly upregulated under salt stress. The high expression levels of these genes conferred salt tolerance in *P. macrophyllus*. Glutathione (GSH) is an essential thiol antioxidant that participates in the detoxification of ROS and improves plant performance under abiotic stress [31]. Previous studies showed that overexpression of the GST gene improved plant growth under salt stress [32–34]. Our study observed that the expression levels of many GST genes were significantly increased following salt stress treatment, and these results are highly consistent with the aforementioned previous reports. Collectively, our results indicated that *P. macrophyllus* rapidly adapted to salt stress at physiological and transcriptomic levels.

### Phytohormone and transcription factor responses to salt stress

Phytohormones are the most important endogenous substances and play a vital role in the regulation of plant growth, development, and acclimation to environmental stimuli. They are also important in modulating the adaptation physiological responses of plants to salt and other abiotic stresses [11, 12]. Following salt stress treatment, the expression levels of genes involved in biosynthetic and polar transport and signaling of multiple hormones in *P. macrophyllus* changed significantly. Genes related to auxin transport and responsiveness, such as *PIN2*, *PIN3*, *AUX1*, *LAXs*, *IAAs*, and *SAURs*, were downregulated in response to salt stress. This result is consistent with a previous study in which the expression of auxin receptor genes was downregulated under salt stress in *Arabidopsis* [35]. The localization of auxin transporters, AUX1 and PINs, changed, and auxin accumulation in roots was reduced in response to salt stress, which decreased auxin signaling to regulate plant growth and enable the plant to adapt to salinity stress [11, 36]. ABA is the most important stress-responsive hormone, and it plays a vital role in plant responses to salinity stress. Overexpression of the ABA receptor-encoding gene (*PYL9*) promoted resistance to osmotic stress in *Arabidopsis* [37]. Our results also showed that the expression levels of *PYL* were upregulated under salt stress in *P. macrophyllus.*

Transcription factors (TFs) are very important in modulating abiotic stress tolerance in plants. Stress-responsive TFs may be important targets for improving abiotic stress tolerance [38]. Many TFs involved in phytohormone signaling pathways, such as basic leucine zipper (bZIP), ABA-responsive element binding factor (ABF), and JA signaling pathways, activate basic helix-loop-helix (bHLH) MYC TFs and cytokinin response regulators, such as *Arabidopsis* response regulator (ARR) 1 and ARR2 [39, 40]. Our RNA-seq data found that three *ARR2* genes were upregulated in *P. macrophyllus* under salt stress, which indicates that cytokinin signaling is also involved in modulating the response to saline stress. MYB TFs constitute a superfamily that is divided into four classes in plants, 1R-, R2R3-, R1R2R3-, and 4R-MYB, and R2R3-MYBs are plant-specific MYB TFs [41]. Numerous R2R3-MYB TFs are involved in primary and secondary metabolism, developmental processes, and responses to biotic or abiotic stresses [41, 42]. *AtMYB44* and its subgroup members (*AtMYB70*, *AtMYB73*, and *AtMYB77*) regulate ABA-mediated stomatal closure in response to abiotic stresses [43]. *AtMYB101*, *AtMYB13*, and *AtMYB15* are involved in ABA-mediated responses to abiotic stress [44]. Our results showed that *MYB73* and *MYB101* were significantly upregulated in *P. macrophyllus* under salt stress. However, other MYB TFs were downregulated, such as *MYB5*-like, *MYB26*, *MYB41*, *MYB52*, *MYB61*, *and MYB86*-like. These TFs are primarily involved in cell wall biogenesis and secondary metabolism in *Arabidopsis*. For example, *AtMYB26* controls secondary wall deposition in anthers [45], and *AtMYB52* and *AtMYB61* regulate cell wall thickening and lignin, xylan, and cellulose biosynthesis [46, 47]. *AtMYB5* regulates trichome development and outer seed coat differentiation [48]. These results are highly consistent with the GO enrichment of downregulated DEGs in our study, which indicates that cell wall organization and/or biogenesis processes were disturbed and retarded in *P. macrophyllus* plants under salt stress. However, this hypothesis requires further study, and the roles of these MYB TFs in salt-tolerance could be tested via CRISPR/Cas mediated genome editing technology [28].

## Conclusion

Short-term and long-term salt stress altered the chloroplast structure of *P. macrophyllus* and significantly reduced chlorophyll content and photosynthetic efficiency. However, the activities of antioxidant enzymes and the concentrations of osmoprotectants in *P. macrophyllus* increased significantly to scavenge ROS and minimize the damage of salinity-induced oxidative stress. At the transcriptome level, a large number of genes involved in metabolic and biological regulation processes were significantly induced by salinity. The genes involved in auxin signaling were downregulated and played critical roles in *P. macrophyllus* adaptation to salt stress, and the genes mediated ABA signaling were upregulated for the general response to salt stress. K^+^ and Ca^2+^ transport and signaling are very important in conferring plant salt tolerance, and many transcription factors, such as MYB, that function in cell wall biosynthesis play important roles. Our study helps elucidate the mechanism of plant tolerance to salt stress and provides a reference for the exploration and use of salt-tolerant genes of *P. macrophyllus* for crop improvement.

## Methods

### Plant material and salt treatments

Seeds of *Podocarpus macrophyllus* var. *angustifolius* were collected from the Xuebaoding National Nature Reserve (30.25 °N, 103.88 °E) in Pingwu County, Sichuan Province, Southwest China. The seeds were germinated in 2017 in a greenhouse. After 3 years, healthy *P. macrophyllus* seedlings of uniform size were moved to a naturally lit glass greenhouse maintained at 30 °C/24 °C and 65% relative humidity. The experimental treatments were performed after 4 weeks, when the seedlings were fully adapted to the environment. A total of 90 plants were used for NaCl treatments at six different concentrations: 0 mM (control, CK), 100 mM, 200 mM, 300 mM, 400 mM, and 500 mM. Each treatment included three biological replicates, with five seedlings per replicate. The plants in the salt-treatment group were watered weekly with 1 L of half-strength Hoagland solution containing the corresponding concentration of NaCl. The fourth to sixth fully expanded leaves of the control group and NaCl-treated group plants were sampled after 3 h and 14 d of treatment for measurements of physiological and biochemical indices and RNA-seq. Each sample had three replicates, and each replicate was a mixture of five seedlings. The collected leaves were immediately frozen using liquid nitrogen and stored at − 80 °C for further experimental analyses.

### Determinations of proline, malondialdehyde (MDA), soluble sugar concentrations, and antioxidant enzyme activities

Proline and MDA concentrations were assayed as described in a previous study [8]. Briefly, fresh leaves (0.5 g) were homogenized in 5 mL of 3% sulfosalicylic acid solution. After centrifugation, 2 mL of supernatant, 2 mL of glacial acetic acid, and 2 mL of 2.5% acid ninhydrin solution were added to a tube. The absorbance (A) of proline in the supernatant was measured at 520 nm, and its concentration is expressed as μg g^− 1^ FW. The absorbance of the samples was measured at 450, 532, and 600 nm using a UV/visible spectrophotometer (GENESYS™ 10S, Thermo Scientific, USA), and the concentration of MDA was calculated using the formula MDA (μmol L^− 1^) = 6.45(A_532_-A_600_)-0.56A_450_. The soluble sugar content was determined using the Lane and Eynon method [49]. Superoxide dismutase (SOD, EC 1.15.1.1) activity was assayed by monitoring the inhibition of photochemical reduction of nitro-blue tetrazolium (NBT) as described by Giannopolitis and Ries [50]. The peroxidase (POD; EC1.11.1.7.) activity was measured at 470 nm, as described by Adam et al. [51]. Catalase (CAT; EC 1.11.1.6.) activity was assayed as described by Kar and Mishra [52].

### Determinations of chlorophyll content, leaf relative water content and biomass, and measurement of chlorophyll fluorescence

Chlorophyll a (Chl *a*) and Chl *b* were extracted with 80% acetone, and their contents were measured using a spectrophotometer at absorbances of 662 nm and 644 nm, respectively, according to the method described by Lichtenthaler et al. [53]. Leaf relative water content (RWC) was determined gravimetrically by weighing leaves before and after oven-drying at 80 °C to a constant mass and expressed as the percentage of water content in dehydrated tissues compared to water-saturated tissues. Chl *a* fluorescence was measured using a portable PAM-2500 chlorophyll fluorometer (Walz, Eichenring, Germany) on the sixth leaf from the apex of *P. macrophyllus* seedlings. The leaves were dark-adapted for 20 min, and a PAR of 900 μmol m^− 2^ s^− 1^ was used for the measurements.

### RNA extraction, cDNA library preparation, and RNA sequencing

Total RNA was extracted from *P. macrophyllus* leaves using TRIzol reagent (Thermo Scientific, USA) according to the manufacturer’s instructions. Each treatment sample had three biological replicates. RNA concentration was measured using a Nanodrop 2000 (Thermo Scientific, USA), and RNA integrity was measured using an Agilent Bioanalyzer 2100 (Agilent Technologies, USA). A total of 2 μg RNA was used for mRNA isolation and RNA-seq library construction using the NEBNext®Ultra™ RNA Library Prep Kit (NEB, USA) according to the manufacturer’s protocol, and index codes were added to attribute sequences to each sample. The cDNA libraries were sequenced at Biomarker Technologies (Beijing, China) on the Illumina NovaSeq 6000 System by 150 bp paired-end sequencing.

### De novo assembly of transcriptome and functional annotation

RNA-seq raw reads were filtered to remove adapter and low-quality bases using Trimmomatic (version 0.36) software. The sequence quality of the RNA-seq data was evaluated using the FastQC (version 0.11.5). De novo assembly using RNA-seq was performed using Trinity (version 2.5.1) software with min_kmer_cov 2 by default and other default parameters [16]. To annotate sequences obtained by de novo assembly, the assembled transcripts were aligned to NCBI non-redundant protein sequences (NR, ftp://ftp.ncbi.nih.gov/blast/db/), Swiss-Prot [54], Gene Ontology (GO) [55], Kyoto Encyclopedia of Genes and Genome (KEGG) [56], Protein family (Pfam) [57], Clusters of Orthologous Groups (COG) [58], the evolutionary genealogy of genes: Non-supervised Orthologous Groups (eggNOG) [59], and EuKaryotic Orthologous Groups (KOG) [24] databases using BLASTX with a significance threshold of E ≤ 1.0 × 10^− 5^.

### Analysis of differentially expressed genes (DEGs)

The expression levels of unigenes were quantified using the R package DESeq2 (version 1.6.3) with parameters for strand-specific RNA-seq [60]. Differentially expressed genes (DEGs) were identified between two comparisons using the following criteria: |log2 (fold change)| > 1 and false discovery rate (FDR) < 0.01. The FDR was generated from an adjusted *p-value* using the Benjamini-Hochberg method. Blast2GO (version 2.5) software was used for enrichment of the GO terms based on the Nr annotation for the non-redundant unigenes, and the KEGG database was used to determine metabolic pathways of the unigenes.

### Transmission Electron Microscopy (TEM) observations

TEM was performed on a section (1–2 mm in length) of the fifth fully expanded leaf of *P. macrophyllus* plants (CK, 3 h, and 14 d after salt treatment) to observe the ultrastructure of chloroplasts according to the procedures described by Han et al. [61]. Leaf sections were fixed with 3% glutaraldehyde (v/v) in 0.1 M phosphate buffer (pH 7.2) for 6 h at 4 °C, followed by 2 h of post-fixation in 1% osmium tetraoxide. Samples were rinsed three times with phosphate buffer (0.1 M, pH 7.2), dehydrated in a graded ethanol series (50, 60, 70, 80, 90, 95, and 100%) and embedded in eponaraldite. Ultrathin sections (80 nm) were sliced, stained with uranyl acetate and lead citrate, and mounted on copper grids for viewing using an H-600IV TEM (Hitachi, Tokyo, Japan).

### Reverse-transcription and quantitative real-time PCR

Total RNA was isolated from the leaves of *P. macrophyllus* using TRIzol reagent (Invitrogen, USA). Four micrograms of total RNA was used to synthesize first-strand cDNA using the PrimeScript RT Reagent Kit (Takara, Japan). Quantitative real-time PCR (qRT-PCR) was performed using CFX (Bio–Rad) real-time PCR equipment with SYBR reagent. The expression level of actin was used as an internal control. Three biological replicates were used for each qRT-PCR analysis. The primer sequences used are listed in Supplementary Table S1.

### Statistical analysis

The data of each group were analyzed separately using SPSS software (version 19.0). Each bar represents the mean ± SE of at least three replicates. Different letters above the bars indicate significant differences, and values of *P* < 0.05 represented statistical significance using Tukey’s test.

## Supplementary Information

**Additional file 1: Supplementary Fig. 1.** Distribution of the different lengths of unigenes in the de novo assembled transcriptome of *P. macrophyllus*. **Supplementary Fig. 2.** Heatmap and Pearson correlation coefficients for RNA-seq replicates of *P. macrophyllus* under SS and LS salt stress and control. Three biological replicates are shown. **Supplementary Fig. 3.** Analysis of the fold changes of the 12 candidate DEGs in *P. macrophyllus* determined by RNA-seq and qRT-PCR. The x-axis represents the comparions between salt stress and CK, the y-axis represents the log2(fold change). **Supplementary Fig. 4.** Percentage of different families of transcription factors that identified in the assembly transcriptome of *P. macrophyllus*. **Supplementary Fig. 5.** The genes which were involved in phytohormones transport or synthesis in the DEGs of *P. macrophyllus* under salt stress. **Supplementary Table 1.** Information of RNA-seq data of *P. macrophyllus*. **Supplementary Table 2.** Primers used for qRT-PCR in *P. macrophyllus*.

**Additional file 2: Supplementary Dataset 1.** Gene co-expression clusters of the upregulated DEGs in *P. macrophyllus* under LS salt stress.

**Additional file 3: Supplementary Dataset 2.** Gene co-expression clusters of the downregulated DEGs in *P. macrophyllus* under LS salt stress.

**Additional file 4: Supplementary Dataset 3.** Transcription factors which were predicted in the assembly transcriptome of *P. macrophyllus*.

## Data Availability

The RNA-seq data produced in this study were deposited in the NCBI Sequence Read Archive (BioProject ID: PRJNA733277; https://dataview.ncbi.nlm.nih.gov/object/PRJNA733277. SRA run accessions: SRR14675926, SRR14675925, SRR14675924, SRR14675920, SRR14675919, SRR14675918, SRR14675923, SRR14675922, SRR14675921). The datasets supporting the conclusions of this article are included within the article and its additional files.

## Abbreviations

ABA: Abscisic acid
ABF: ABA-responsive element binding factor
APX: Ascorbate peroxidase
ARR: Arabidopsis response regulator
bHLH: Basic helix-loop-helix
bZIP: Basic leucine zipper
CaMs: Calmodulin-related proteins
CAT: Catalase
CK: Control
CMLs: Calcium-binding proteins
COG: Clusters of Orthologous Groups
DEGs: Differentially expressed genes
eggNOG: Evolutionary genealogy of genes: Non-supervised Orthologous Groups
GA: Gibberellin
GO: Gene Ontology
GST: Glutathione S-transferase
HKT1: High affinity potassium Transporter1
JA: Jasmonates
KEGG: Kyoto Encyclopedia of Genes and Genomes
KOG: EuKaryotic Orthologous Groups
LRW: Leaf relative water
LS: Long-term salt treatment
MDA: Malondialdehyde
PCA: Principal component analysis
POD: Peroxidase
qRT-PCR: Quantitative real-time Polymerase Chain Reaction
ROS: Reactive oxygen species
RNA-seq: RNA sequencing
SA: Salicylic acid
SOD: Superoxide dismutase
SOS1: Salt Overly Sensitive 1
SS: Short-term salt treatment
TFs: Transcription factors
TPS: Trehalose-6-phosphatase synthase
XYLs: Alpha-xylosidase
