# Supplementary material for: De novo transcriptome analysis provides insights into the salt tolerance of Podocarpus macrophyllus under salinity stress
Source: BMC Plant Biol. 2021 Oct 25;21:489. doi: 10.1186/s12870-021-03274-1 (PMC8543971; doi:10.1186/s12870-021-03274-1)
Supplement: Supplementary file 1 — Additional file 1: Supplementary Fig. 1. Distribution of the different lengths of unigenes in the de novo assembled transcriptome of P. macrophyllus. Supplementary Fig. 2. Heatmap and Pearson correlation coefficients for RNA-seq replicates of P. macrophyllus under SS and LS salt stress and control. Three biological replicates are shown. Supplementary Fig. 3. Analysis of the fold changes of the 12 candidate DEGs in P. macrophyllus determined by RNA-seq and qRT-PCR. The x-axis represents the comparions between salt stress and CK, the y-axis represents the log2(fold change). Supplementary Fig. 4. Percentage of different families of transcription factors that identified in the assembly transcriptome of P. macrophyllus. Supplementary Fig. 5. The genes which were involved in phytohormones transport or synthesis in the DEGs of P. macrophyllus under salt stress. Supplementary Table 1. Information of RNA-seq data of P. macrophyllus. Supplementary Table 2. Primers used for qRT-PCR in P. macrophyllus. [file 12870_2021_3274_MOESM1_ESM.pdf]

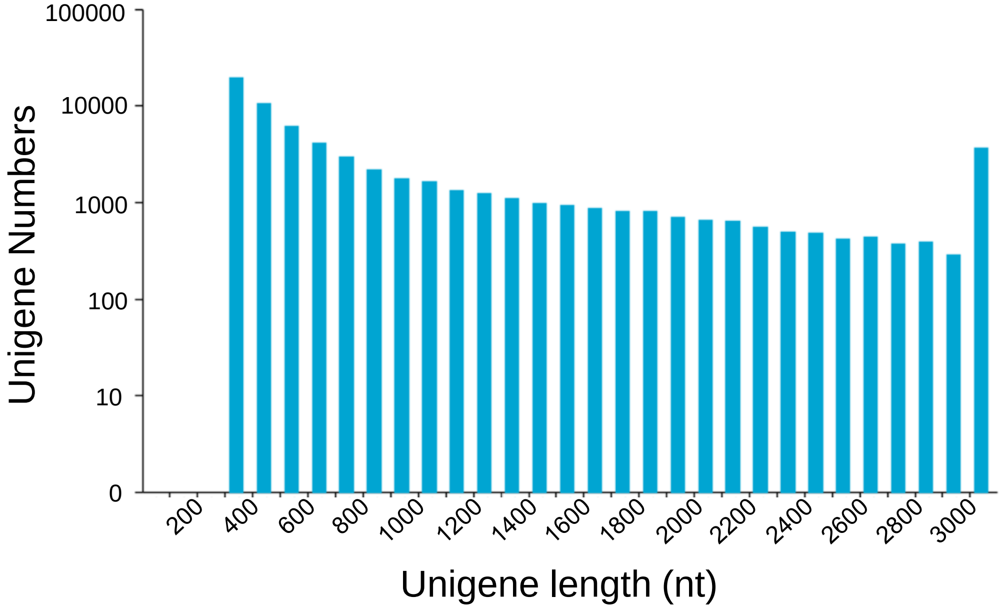

Supplementary Figure 1. Distribution of the different lengths of unigenes in the *de novo* assembled transcriptome of *P. macrophyllus*.

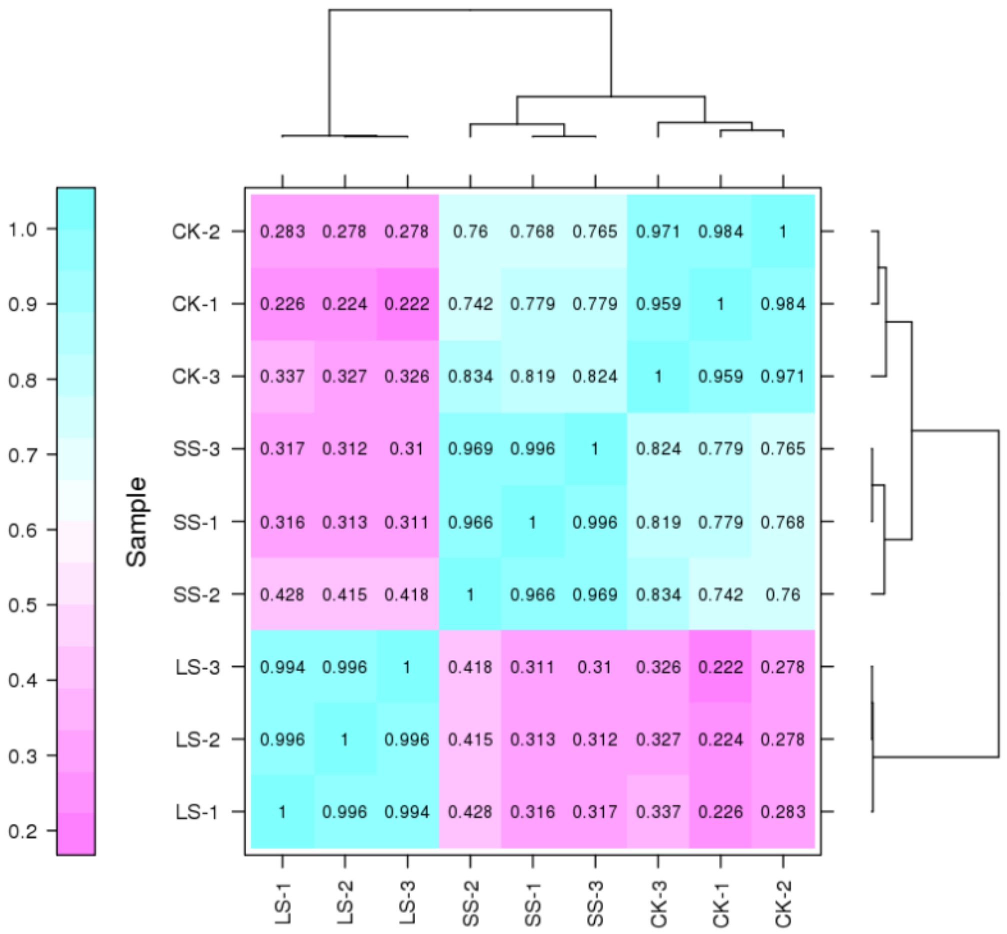

Supplementary Figure 2. Heatmap and Pearson correlation coefficients for RNA-seq replicates of *P. macrophyllus* under SS and LS salt stress and control. Three biological replicates are shown.

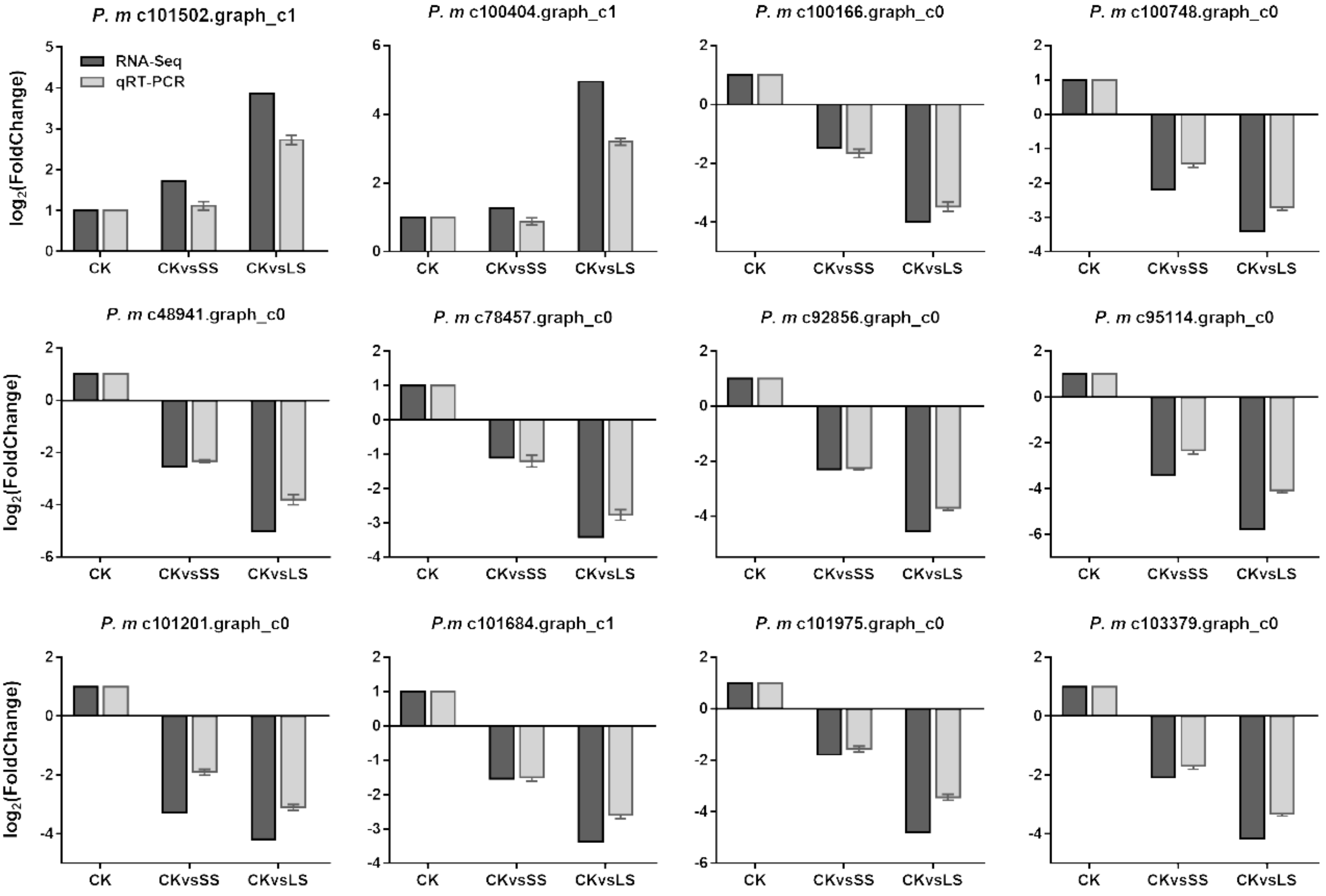

Supplementary Figure 3. Analysis of the fold changes of the 12 candidate DEGs in *P. macrophyllus* determined by RNA-seq and qRT-PCR. The x-axis represents the comparions between salt stress and CK, the y-axis represents the log2(fold change).

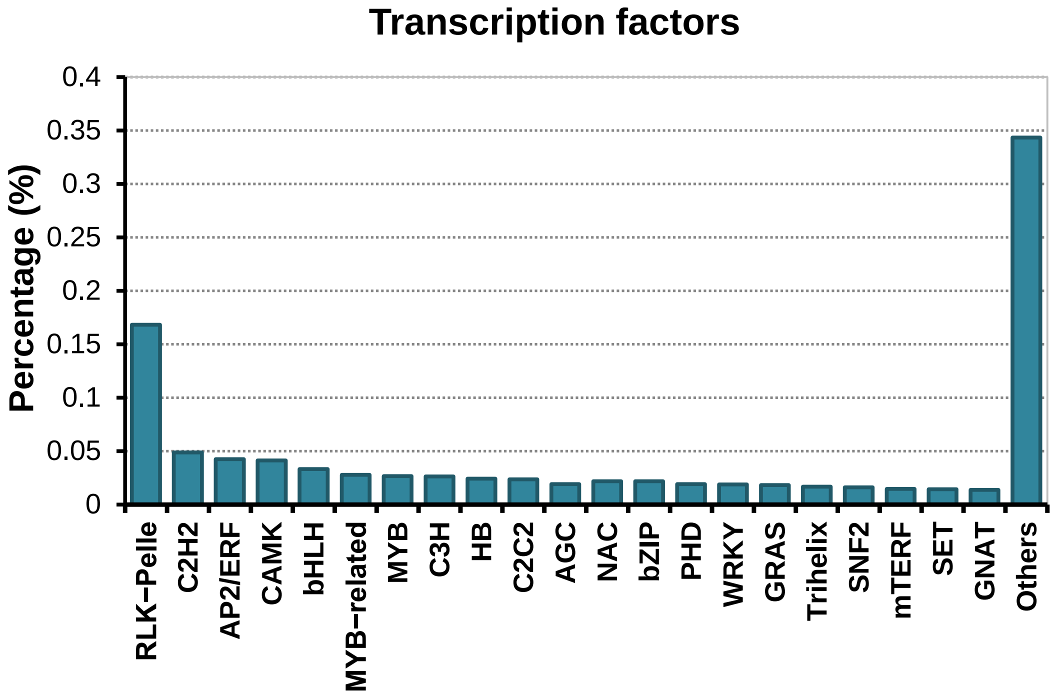

Supplementary Figure 4. Percentage of different families of transcription factors that identified in the assembly transcriptome of *P. macrophyllus*.

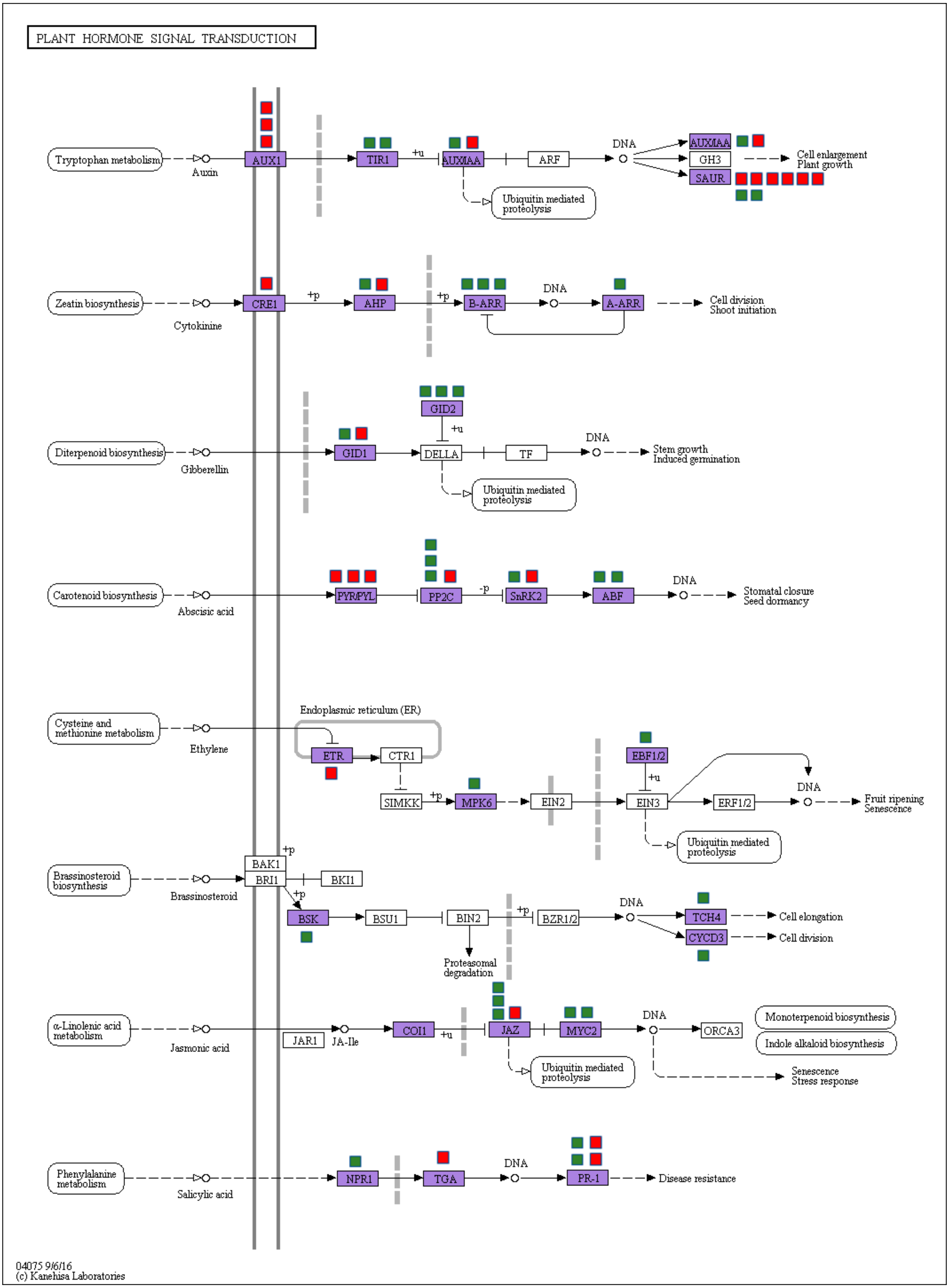

Supplementary Figure 5. The genes involved in phytohormones transport or synthesis in the DEGs of *P. macrophyllus* under salt stress.

Supplementary Table 1. Information of RNA-seq data of *P. macrophyllus*.

Samples	Replicate	Clean Reads	Base Number	Mapped reads	Mapped Ratio	GC Content	% ≥ Q30
Control	1	22,977,316	6,883,571,484	19,466,660	84.72%	45.68%	94.56%
Control	2	23,184,133	6,940,680,172	19,736,403	85.13%	45.36%	94.48%
Control	3	25,143,716	7,536,426,190	21,795,599	86.68%	45.50%	94.49%
Salt-3h	1	25,018,533	7,490,966,990	21,334,701	85.28%	45.35%	94.27%
Salt-3h	2	22,717,951	6,804,419,372	19,333,906	85.10%	45.55%	94.86%
Salt-3h	3	24,819,231	7,429,970,482	21,250,331	85.62%	45.54%	95.02%
Salt-14d	1	23,264,042	6,968,409,636	19,269,476	82.83%	45.80%	94.42%
Salt-14d	2	25,203,205	7,544,301,178	20,621,413	81.82%	46.00%	94.49%
Salt-14d	3	25,253,988	7,566,205,914	20,523,535	81.27%	45.91%	94.40%

Supplementary Table 2. Primers used for qRT-PCR in *P. macrophyllus*.

Primers name	Sequences (5'-3')
P.m c100166.graph_c0-F	ATCTCGGTCGGGATAGGA
P.m c100166.graph_c0-R	TATTCAAAGACGAAGGTGGT
P.m c95114.graph_c0-F	GTATGGCAGTATGATCGTGTT
P.m c95114.graph_c0-R	GAGCGAATTGCTTGGTCT
P.m c100748.graph_c0-F	AGCCAAAGAAGCCCAAGA
P.m c100748.graph_c0-R	CAAATCAAACGCAAACCTC
P.m c101201.graph_c0-F	CCCCAACACTAACAGGATG
P.m c101201.graph_c0-R	CGGTGACAGTACCCAAGC
P.m c101684.graph_c0-F	TGCGTGTTGGATTGTAGC
P.m c101684.graph_c0-R	AATGGCAAACTCGTTATGG
P.m c101975.graph_c0-F	GGGTAAGAGTATTAAGCCACA
P.m c101975.graph_c0-R	AATAAAGATTGCTGGAAACAG
P.m c103379.graph_c0-F	CGCCTACACCCATAACTTAG
P.m c103379.graph_c0-R	AGCAGCCTCACTCCTCTG
P.m c48941.graph_c0-F	TCGGGAAGGGCAACCA
P.m c48941.graph_c0-R	CACCGCCACGACGATAT
P.m c78457.graph_c0-F	GTTGAAATTCATGGGCAATA
P.m c78457.graph_c0-R	TCCTCCTCCCTTACCACC
P.m c92856.graph_c0-F	AGAGGCACGAATGAAAGTC
P.m c92856.graph_c0-R	ATAAGCGTCAGTAACAAACATG
P.m c100404.graph_c1-F	AAATAGTTTCCCTCCTTGATTT
P.m c100404.graph_c1-R	GTGAATTGCCGTCCCTCT
P.m c101502.graph_c1-F	ACGCTCATCCACCTTACAAC
P.m c101502.graph_c1-R	GAGCTCTGATCCTTCTCCC
Actin-F	TTGCTCCAGAAGAACATC
Actin-R	ATGGCAACATACATAGCAGG
